# A *Lindera obtusiloba* Extract Blocks Calcium-/Phosphate-Induced Transdifferentiation and Calcification of Vascular Smooth Muscle Cells and Interferes with Matrix Metalloproteinase-2 and Metalloproteinase-9 and NF-**κ**B

**DOI:** 10.1155/2015/679238

**Published:** 2015-07-30

**Authors:** Christian Freise, Ki Young Kim, Uwe Querfeld

**Affiliations:** ^1^Department of Pediatric Nephrology and Center for Cardiovascular Research, Charité-University Medicine, Campus Virchow Clinic, 10115 Berlin, Germany; ^2^Department of Beauty Design, Human Environmental Sciences College, Wonkwang University, Iksan 570-749, Republic of Korea

## Abstract

Vascular calcifications bear the risk for cardiovascular complications and have a high prevalence among patients with chronic kidney disease. Central mediators of vascular calcifications are vascular smooth muscle cells (VSMC). They transdifferentiate into a synthetic/osteoblast-like phenotype, which is induced, for example, by elevated levels of calcium and phosphate (Ca/P) due to a disturbed mineral balance. An aqueous extract from* Lindera obtusiloba* (LOE) is known to exert antifibrotic and antitumor effects or to interfere with the differentiation of preadipocytes. Using murine and rat VSMC cell lines, we here investigated whether LOE also protects VSMC from Ca/P-induced calcification. Indeed, LOE effectively blocked Ca/P-induced calcification of VSMC as shown by decreased VSMC mineralization and secretion of alkaline phosphatase. In parallel, mRNA expression of the calcification markers osterix and osteocalcin was reduced. Vice versa, the Ca/P-induced loss of the VSMC differentiation markers alpha smooth muscle actin and smooth muscle protein 22-alpha was rescued by LOE. Further, LOE blocked Ca/P-induced mRNA expressions and secretions of matrix metalloproteinases-2/-9 and activation of NF-*κ*B, which are known contributors to vascular calcification. In conclusion, LOE interferes with the Ca/P-induced transdifferentiation/calcification of VSMC. Thus, LOE should be further analysed regarding a potential complementary treatment option for cardiovascular diseases including vascular calcifications.

## 1. Introduction

Among patients with chronic kidney disease (CKD), cardiovascular complications are the leading cause of death [1]. Thus, most CKD patients die because of a systemic damage of the cardiovascular system. The formation of vascular calcifications in vessels or the heart plays an important role and is of vital importance for the life expectancy of dialysis patients [2]. During the onset of arterial calcifications, vascular smooth muscle cells (VSMC) in the media transdifferentiate from a quiescent/contractile phenotype to a synthetic/osteoblast-like phenotype [3]. As of today, there is no established therapy for the inhibition of onset and progression of vascular calcification.

Compositions or mixtures of medicinal plants are traditionally used to prevent or treat certain diseases like cancer or liver diseases [4, 5]. In traditional Chinese and Korean medicine, preparations from the medicinal plant* Lindera obtusiloba* are applied to treat inflammations and to improve blood circulation [6]. A drinkable aqueous extract from* Lindera obtusiloba* (LOE) comprises a good physiological compatibility and was previously shown to exert beneficial effects in models of adipogenesis, liver fibrosis, and liver cancer [7–9]. So far, identified bioactive compounds from LOE are, for example, polyphenols like (+)-episesamin [10], the antioxidant compound quercitrin [11], and substances which belong to the group of lignans and butenolides [12, 13].

The traditional effects on blood circulation [6] along with antioxidative and anti-inflammatory effects of LOE [7–9] led us to the hypothesis that LOE might exert preventive effects also in models of vascular calcification.

To investigate this issue, we used an established* in vitro* model of vascular calcification which enables the induction of calcification/transdifferentiation of VSMC with elevated concentrations of calcium and phosphorus (Ca/P) [14].

## 2. Materials and Methods

### 2.1. Preparation and Standardization of the* Lindera obtusiloba* Extract

Freeze-dried extracts of* Lindera obtusiloba* were obtained as described previously [9]. To obtain stock solutions, 10 mg powder was dissolved in 10 mL sterile distilled water at 60°C for 30 min. Aliquots were stored at −20°C. Freshly prepared working solutions of* L. obtusiloba* extract were routinely tested/standardized as described [8, 9] before being used in the respective calcification experiments.

### 2.2. Cell Culture

The murine VSMC cell lines (MOVAS-1; ATCC CRL-2797 and human aortic VSMC) (Life Technologies, Karlsruhe, Germany) were cultured in a humidified atmosphere at 37°C and 5% CO_2_. Standard culture medium consisted of DMEM with 862 mg/L L-alanyl-L-glutamine, 1.0 g/L glucose, 50 *µ*g/mL streptomycin, 50 units/mL penicillin, and 0.2 mg/mL G418, supplemented with 10% heat-inactivated fetal bovine serum (FBS; Biochrom, Berlin, Germany). Cell layers were detached with 0.05% trypsin/0.02% EDTA solution (Biochrom). Cells were maintained at 70–80% confluence by passaging as needed. Human VSMC were used from passages 5 to 12.

### 2.3. Induction of VSMC Calcification

VSMC were grown in 24-well plates (Becton Dickinson, Heidelberg, Germany) until they were ~90% confluent (day 0). The culture medium was then replaced by calcification medium (Ca/P) which consists of standard culture medium supplemented with calcium chloride (2.7 mM versus 1.8 mM) and sodium dihydrogen phosphate (2.8 mM versus 1.0 mM). VSMC were then cultured under the different treatment conditions for 9-10 days as indicated. Media were replaced every second day.

### 2.4. Analysis of VSMC Calcification

At the end of the treatment, the cells were washed three times with calcium-free PBS and decalcified with 0.1 M HCl for 30 min. Calcium content in the supernatant was determined by the o-cresolphthalein complexone (OCPC) method as described [14]. Calcium contents were normalized to the protein contents of the lysates which were determined by the BCA method according to the manufacturer's instructions (Thermo Fisher Scientific, Waltham, MA, USA).

In addition, alizarin red staining of mineralized VSMC was performed. At the end of the treatment, the cells were fixed with 4% paraformaldehyde for 10 min and washed with distilled water before the incubation with 2% alizarin red solution. After 30 min on a rotary shaker, wells were washed three times with equal volumes of distilled water. Photographs were taken by a digital microscope (Keyence, Neu-Isenburg, Germany). Alizarin red staining was quantified by resolving bound alizarin red dye with 2.5% cetylpyridinium chloride and photometric measurement of absorbance at 540 nm using an ELISA reader (Biorad, Munich, Germany).

As a characteristic marker of vascular calcification, the content of alkaline phosphatase (ALP) in VSMC supernatants was analyzed with the Quanti Blue reagent (InvivoGen, San Diego, CA, USA) as described [14].

### 2.5. Quantitative Real-Time Reverse Transcription-Polymerase Chain Reaction (RT-PCR)

VSMC in 6-well tissue culture plates at ~90% confluency were serum starved for 24 h in standard culture medium containing 0.2% FBS. Cells were then treated for 24 h as indicated. Total RNA was isolated and transcribed into complementary DNA (cDNA) with the RNeasy Mini Kit (Quiagen, Hilden, Germany) and the High Capacity RNA to DNA kit (Applied Biosystems, Foster City, CA, USA), respectively. Quantitative PCR using SYBR Green Master Mix (Applied Biosystems) was performed on MxPro-system from Stratagene (Agilent Technologies, Santa Clara, CA, USA). The relative amount of RNA was calculated using the 2^−ΔΔCt^ method and normalized to mRNA expressions of the housekeeping genes YWAHZ (tyrosine 3-monooxygenase/tryptophan 5-monooxygenase activation protein, zeta polypeptide) or HPRT (hypoxanthine phosphoribosyltransferase). The primer sequences used are available on demand. All measurements were performed in triplicate.

### 2.6. Measurement of Effects of LOE on VSMC Viability

Cell cycle synchronized VSMC were treated with different concentrations of LOE for 24–48 h and viable cells were detected as described by conversion of the fluorescent dye calcein AM by intracellular esterase [7].

### 2.7. Determination of MMP-Activity in VSMC Supernatants

Enzymatic activities of MMP-2 and MMP-9 were measured by cleavage of 0.01 mg/mL dye-quenched DQ-gelatin (Molecular Probes) as described [15]. Cells were treated as mentioned above for calcification. At the indicated time points, the medium was replaced by serum-free growth medium for 16 h. 50 *μ*L of the supernatants was transferred to a black 96-well microtiter plate with clear bottom (Greiner Bio-One). Each well received 150 *μ*L DQ-gelatin solution and fluorescence signals were monitored for 140 min at 37°C using a Victor^3^ microplate reader (Perkin Elmer). Background subtraction was applied to all measurements.

### 2.8. Measurement of NF-*κ*B Activation in VSMC

For the luciferase reporter assay experiments, VSMC were transiently transfected in white 96-well plates (NUNC, Roskilde, Denmark) with reporter plasmids for the NF-*κ*B pathway (pGL4.32[luc2P/NF-*κ*B-RE/Hygro]) and a Renilla vector (pGL4.74[hRluc/TK]) for normalization of transfection efficiencies. Transfections were performed using the FuGENE HD Transfection Reagent (Promega, Mannheim, Germany) according to the manufacturer's instructions. 24 h after transfection, the cells were treated as indicated for 24 h and analysed using the Dual-Glo Luciferase Assay System (Promega) according to the manufacturer's instructions using a Victor^3^ microplate reader (Perkin Elmer).

### 2.9. Statistical Analyses

One-way ANOVA followed by the Tukey* post hoc* test was used to analyse the datasets using GraphPad PRISM, version 6.01 (GraphPad Software, USA), and differences with *P* values ≤0.05/≤0.01/≤0.001  (*∗*/*∗∗*/*∗∗∗*) were considered to be statistically significant.

## 3. Results

### 3.1. LOE Attenuates Ca/P-Induced Calcification in VSMC

**Figure 1:**
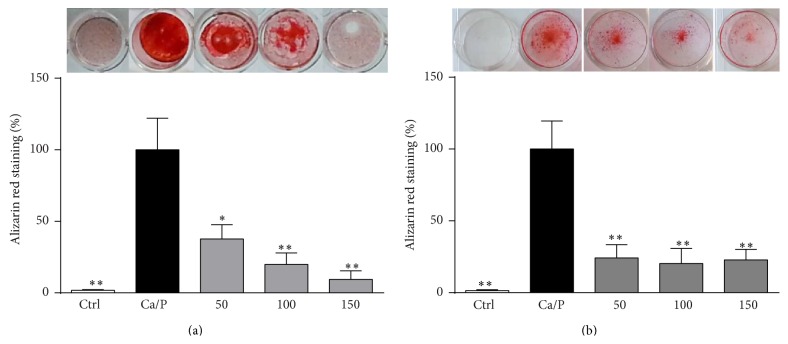
LOE attenuates Ca/P-induced VSMC mineralization. VSMC were cultured as indicated for 9 d. Shown are representative images of alizarin red stained murine (a) and human (b) VSMC together with the respective quantification of bound alizarin red dye from four independent experiments. Shown are means ± SD. ^*∗*^ *P* ≤ 0.05, ^*∗∗*^ *P* ≤ 0.01.

VSMC treated with standard culture medium (1.03 mM PO_4_^3−^; 1.0 mM Ca^2+^) exhibited no calcifications whereas VSMC treated with calcification medium (2.8 mM PO_4_^3−^; 2.7 mM Ca^2+^; Ca/P) showed a strong degree of calcification after 9 d as shown by enhanced alizarin red staining in murine (Figure 1(a)) and human VSMC (Figure 1(b)), respectively. In the presence of LOE these calcifications were distinctly decreased.

**Figure 2:**
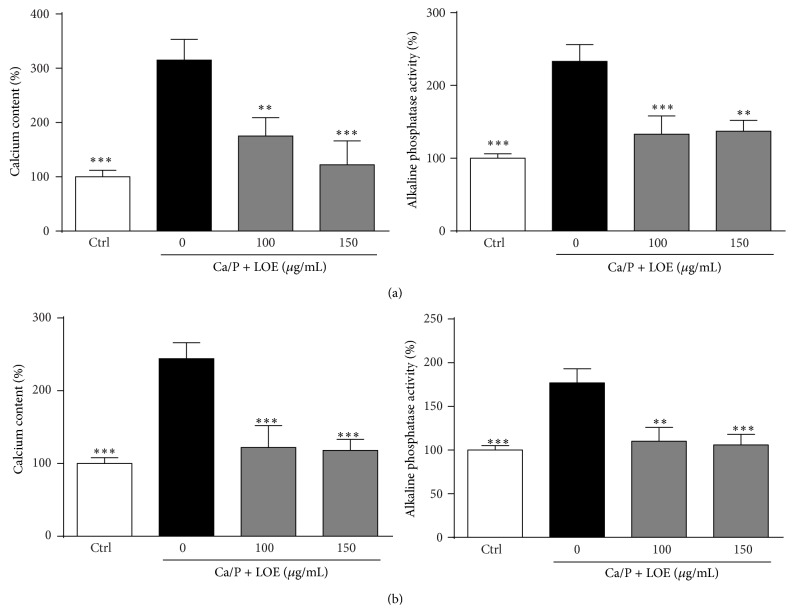
Effects of LOE on Ca/P-induced VSMC calcification and production of alkaline phosphatase. Calcium contents and alkaline phosphatase (ALP) amounts in supernatants served as additional markers of VSMC calcification. Shown are treatment dependent effects on calcium and ALP levels in murine (a) and human VSMC (b) (*n* = 4; mean ± SD; ^*∗∗*^ *P* ≤ 0.01, ^*∗∗∗*^ *P* ≤ 0.001).

These data were confirmed by measurements of calcium amounts in the respective VSMC cultures and the release of alkaline phosphatase (ALP) into the VSMC supernatants. While the calcification medium caused a strong upregulation of both parameters in murine (Figure 2(a)) and human VSMC (Figure 2(b)), LOE almost neutralized the stimulating effects of the calcification medium on calcium accumulation and ALP secretion.

### 3.2. The Attenuating Effects of LOE on VSMC Calcification Are Not due to Cytotoxicity

**Figure 3:**
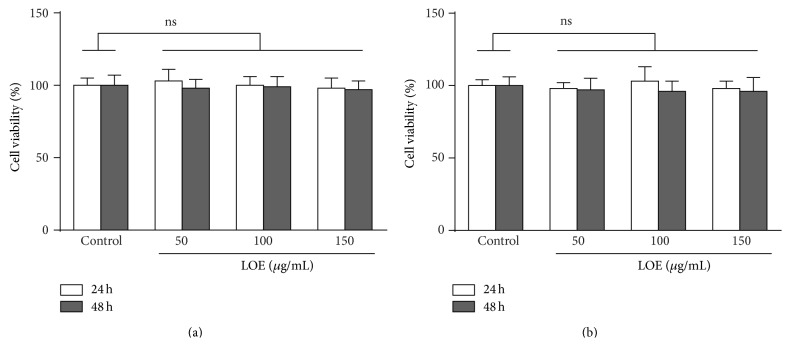
Effects of LOE on the viability of VSMC. Cell viability of murine (a) and human (b) VSMC was quantified fluorometrically after 24–48 h treatment with different concentrations of LOE by intracellular esterase-mediated conversion of calcein AM and was calculated in relation to vehicle treated cells (100%). Shown are means ± SD (*n* = 3; ^ns^not significant).

The preceding data showed that LOE reduced calcification in VSMC. To ensure that these effects are not due to cytotoxic effects, equal LOE concentrations were tested in cytotoxicity assays in VSMC.  Figure 3 shows that even the highest LOE concentration of 150 *µ*g/mL did not significantly affect the viability of murine (Figure 3(a)) and human VSMC (Figure 3(b)).

### 3.3. LOE Blocks Calcification Medium-Induced mRNA Expressions of Osterix and Osteocalcin in VSMC

**Figure 4:**
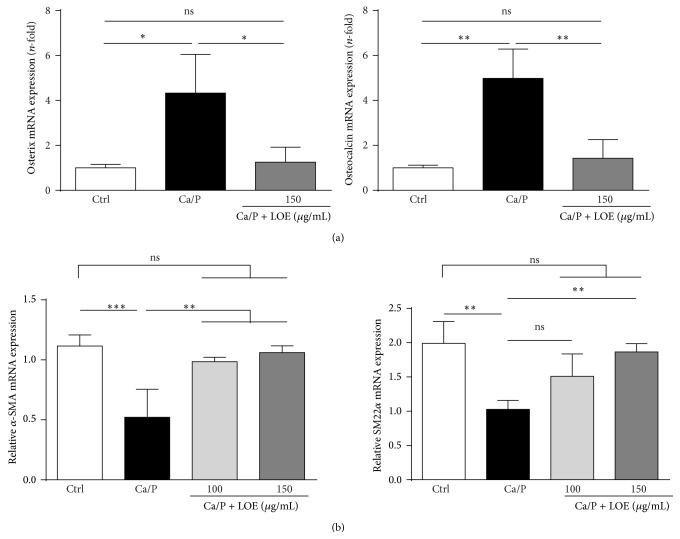
Effects of LOE on Ca/P-induced mRNA expressions of osterix, osteocalcin, *α*-SMA, and SM22*α*. The mRNA expressions of osterix and osteocalcin (*n* = 3) (a) and of *α*-SMA and SM22*α* (*n* = 4) (b) in murine VSMC were determined by RT-PCR. The expression pattern after 24 h of stimulation is shown (means ± SD; ^ns^not significant; ^*∗*^ *P* ≤ 0.05; ^*∗∗*^ *P* ≤ 0.01; ^*∗∗∗*^ *P* = 0.001).

To gain insights into processes on molecular levels, treatment dependent changes in mRNA expression levels of the typical calcification markers osterix and osteocalcin were determined in murine VSMC. As shown in Figure 4(a), the calcification medium induced a strong upregulation of osterix and osteocalcin mRNA expressions, which was attenuated by cotreatment with LOE. In addition, we investigated effects of LOE on the expression levels of typical markers of the differentiated, contractile VSMC phenotype. As expected, Ca/P-treated VSMC exhibited distinctly reduced expression levels of alpha smooth muscle actin (*α*-SMA) and smooth muscle protein 22-alpha (SM22*α*) (Figure 4(b)). However, cotreatment with 150 *µ*g/mL LOE prevented VSMC from reduced expressions levels of *α*-SMA and SM22*α* (Figure 4(b)).

### 3.4. LOE Attenuates Ca/P-Induced mRNA Expressions of MMP-2 and MMP-9 and Gelatinolytic Activities in VSMC Supernatants

**Figure 5:**
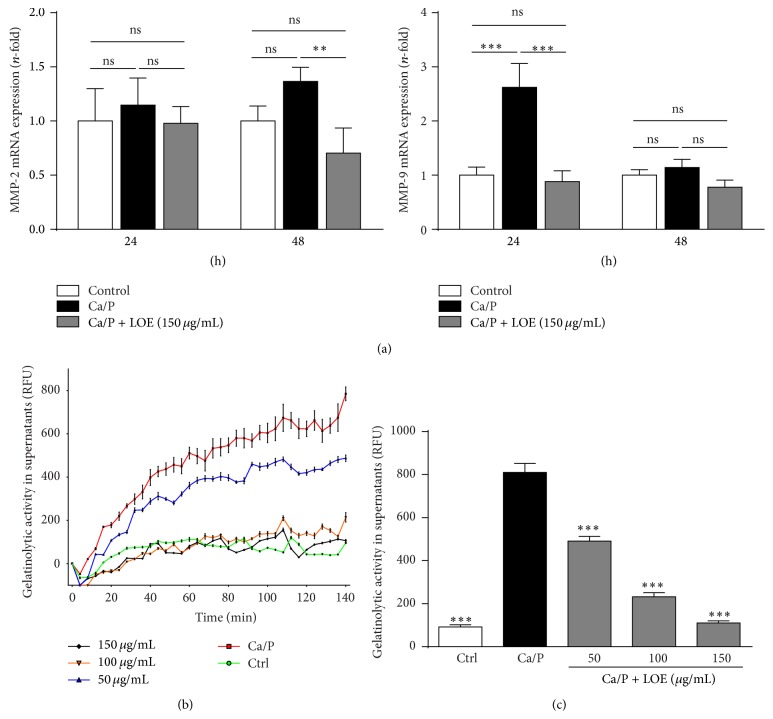
Effects of LOE on Ca/P-induced mRNA expression of MMP-2 and MMP-9 and gelatinolytic activities in VSMC supernatants. (a) Shown are the treatment dependent mRNA expression levels of MMP-2 and MMP-9 in murine VSMC after 24–48 h (*n* = 3). (b) Shown is a representative result of one measurement of gelatinolytic activities in VSMC supernatants by cleavage of DQ-gelatin and (c) the corresponding means ± SD of the respective endpoints of three independent measurements (^ns^not significant; ^*∗∗*^ *P* ≤ 0.01, ^*∗∗∗*^ *P* ≤ 0.001).

Enhanced proteolytic activity by matrix metalloproteinases has been shown to promote vascular calcification and LOE is known to exert inhibitory effects on MMPs. We therefore analysed if LOE impacts the Ca/P-induced expression of the gelatinases MMP-2 and MMP-9 by murine VSMC. As expected, Ca/P-treated VSMC exhibited mRNA upregulations of both MMPs after 24–48 h (Figure 5(a)). In contrast to the MMP-2 mRNA expression which shows a slight and steady increase at 24 h and 48 h, the mRNA expression of MMP-9 reaches a maximum after 24 h and is almost reduced to the basal level after 48 h. In the presence of LOE, the Ca/P-induced increased mRNA expressions of MMP-2 as well as of MMP-9 were neutralized to the expression level of the control at each time point.

In parallel, experiments which detect treatment dependent gelatinolytic activities in supernatants from VSMC were performed. LOE distinctly reduced the elevated gelatinolytic activities in aliquots of serum-free supernatants which were incubated with the differently treated VSMC.  Figure 5(b) shows one representative result out of three experiments and Figure 5(c) shows a summary of respective endpoints from three independent experiments.

### 3.5. LOE Reduces the Activation of the Transcription Factor NF-*κ*B by Ca/P in VSMC

**Figure 6:**
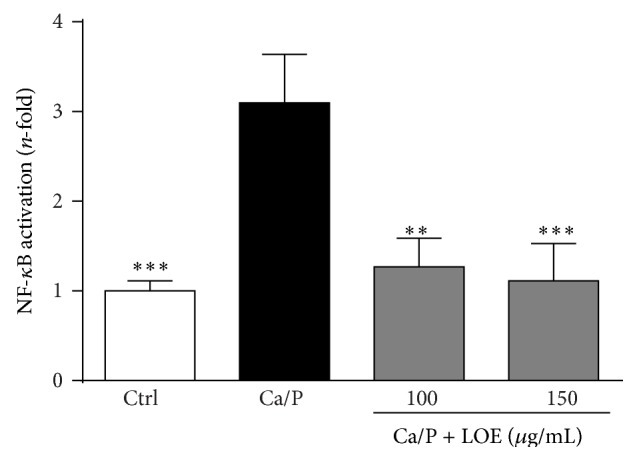
Effects of LOE on Ca/P-induced activation of NF-*κ*B in VSMC. Treatment dependent changes in luciferase activity as an indicator for activation or inhibition of NF-*κ*B were detected in murine VSMC after 24 h. Shown are means ± SD of three independent measurements. ^*∗∗*^ *P* ≤ 0.01, ^*∗∗∗*^ *P* ≤ 0.001.

The expression levels of MMPs in VSMC are regulated by the transcription factor NF-*κ*B. We therefore studied if LOE is capable of decreasing the activation of NF-*κ*B by Ca/P. As shown in Figure 6, LOE indeed effectively blocked an increase of NF-*κ*B activation after treatment of murine VSMC with the calcification medium.

## 4. Discussion

We here provide evidence that an aqueous extract from the medicinal plant* Lindera obtusiloba* (LOE) protects VSMC from artificial induced calcifications by a calcification medium (containing elevated levels of calcium and phosphorus).

A disturbed balance of minerals is a common feature of cardiovascular diseases associated with CKD. Elevated plasma levels of calcium and phosphorus contribute to the induction of VSMC transdifferentiation which represents an underlying step in the development of vascular calcifications.

To screen potential inhibitors of vascular calcification, this transdifferentiation of VSMC can be mimicked or induced* in vitro* by elevated concentrations of calcium and phosphorus [16]. In our experiments, the calcification medium-treated VSMC exhibited strong signs of calcification as demonstrated by enhanced alizarin red staining and elevated calcium contents in VSMC cultures and also elevated levels of alkaline phosphatase in VSMC supernatants. All these effects were strongly reduced by cotreatment with LOE. This clearly suggests protective effects of LOE on calcification of VSMC.

On a molecular level, an early step of VSMC transdifferentiation is the upregulation of bone differentiation markers which are commonly used as markers to indicate the phenotypic conversion of VSMC to osteoblast-like cells [17–21]. As shown here, LOE effectively decreased calcification medium-induced upregulation of osterix and osteocalcin mRNA expression. In conjunction with the here shown protective effects of LOE against Ca/P-induced loss of mRNA expressions of *α*-SMA and of SM22*α*, our data indicate that LOE prevents VSMC from calcium-/phosphate-induced transdifferentiation, thereby preventing VSMC calcifications.

As previously also shown in other cell types [7, 9], LOE decreased the mRNA expression and enzymatic activity of the gelatinases MMP-2 and MMP-9 in VSMC. Both MMPs were shown to promote vascular calcifications associated with CKD [22, 23]. They are believed to induce proteolytic remodelling of the extracellular (vessel) matrix and thereby are supposed to generate biologically active matrix fragments which might amplify the process of VSMC calcification [24, 25]. These effects of MMPs on vascular calcification could be blocked by pharmacological inhibition of MMPs [26, 27]. LOE was previously shown to reduce the secretion of gelatinases by preadipocytes and the enzymatic activity of recombinant MMP-2 [9]. In combination with the here shown interference of LOE with Ca/P-induced transcription in VSMC and gelatinolytic activity in VSMC supernatants, these data suggest that MMPs represent an important target of LOE in the inhibition of VSMC calcification.

The activation of the transcription factor NF-*κ*B is involved in the regulation of MMP-production [28, 29] and also VSMC calcification [30]. We here show that LOE blocks the Ca/P-induced activation of NF-*κ*B in VSMC and also slightly reduces its basal activity. On the one hand, this might explain the observed effects of LOE on MMP-mRNA expressions of MMP-2 and MMP-9 and on the other hand might also directly contribute to attenuating effects of LOE on VSMC calcification. The latter assumption could be deduced from experiments where the NF-*κ*B inhibitor 17-DMAG also directly attenuated Ca/P-induced calcifications of VSMC [14].

Several studies deal with the isolation and structural characterization of the active constituents from* Lindera obtusiloba* [11, 12, 31]. In our group, we previously isolated and identified the lignan (+)-episesamin in LOE [10]. A recent study suggests that (+)-episesamin exerts protective effects in VSMC associated cardiovascular diseases [32] and is therefore a suitable candidate for further studies in models of vascular calcification.

But irrespective of additional, potentially active, components contained in LOE, the extract itself should also be tested in animal models of vascular calcification. Herbal extracts often contain several groups of biologically active compounds and it is not always possible to know which compound is responsible for the observed activities. In addition, different groups of compounds in plant extracts may have synergistic effects. Therefore, besides analyzing the composition and identification of single drugs in LOE, also further testing of the whole extract seems indicated.

## 5. Conclusion

In conclusion, our study demonstrates the potential of LOE to impact the pathophysiology of vascular calcification by affecting VSMC transdifferentiation and to inhibit MMPs and NF-*κ*B in VSMC. Regarding the development of novel complementary therapeutic approaches for cardiovascular diseases, further studies with LOE or isolated drugs from LOE should be performed.
